# Influence Mechanism of Confining Pressure on Morphology of Concrete Crack Surfaces

**DOI:** 10.3390/ma18133158

**Published:** 2025-07-03

**Authors:** Yuqiang He, Chenyang Zhao, Zhiming Xiao, Mingfeng Lei, Chaojun Jia

**Affiliations:** 1 China Energy Shuohuang Railway Development Co., Ltd., Cangzhou 062350, China; 2 School of Mechanics and Civil Engineering, China University of Mining and Technology, Xuzhou 221116, China; 3 School of Civil Engineering, Central South University, Changsha 410075, China; 4 MOE Key Laboratory of Engineering Structure of Heavy Haul Railway, Changsha 410075, China

**Keywords:** characteristics of concrete crack surfaces, fractal dimension, triaxial compression, 3D laser scanning

## Abstract

Characterizing the morphological characteristics of concrete crack surfaces is fundamental for accurately analyzing the evolution mechanism of leakage in concrete linings. In this research, concrete crack surfaces are obtained using triaxial compression tests and three-dimensional (3D) laser scanning. The mechanism by which confining pressure influences crack morphology is further revealed, and the impact of crack morphology on tunnel lining leakage is illustrated from the perspective of fractal dimensions. The results indicate that the concrete crack surface flattens as the confining pressure increases. The distribution of asperity on concrete crack surfaces exhibits strong randomness. A negative exponential function can effectively depict the relationship between the fractal dimension of a concrete crack surface and the confining pressure. As the fractal dimension decreases with increasing confining pressure, concrete cracks developing under higher confining pressure exhibit a higher permeability coefficient, and tunnel linings become more susceptible to water leakage.

## 1. Introduction

Water leakage is one of the key concerns in engineering fields such as tunnels and underground chambers [1,2,3,4]. The global concrete restoration market, valued at USD 18.6 billion in 2024, is projected to expand to USD 29.3 billion by 2030 at an 8.3% compound annual growth rate [5]. This robust growth trajectory highlights the escalating severity of water infiltration in aging concrete infrastructure. The occurrence of water leakage mainly stems from cracks in concrete linings. Cracks not only serve as pathways for seepage but also impact water flow within concrete linings through their morphological characteristics [6,7]. To avoid the adverse impact of water leakage on the operational safety of underground structures, some works, such as predicting water leakage volume and formulating relevant prevention and control measures, can be carried out once the evolution mechanism of water leakage is revealed. It is therefore necessary to characterize the morphological characteristics of concrete crack surfaces in order to reveal the mechanisms behind water leakage evolution.

Existing research on the morphological characteristics of concrete crack surfaces has considered material types [8,9,10], loading patterns [11,12], curing conditions [13,14,15], and service environments [16,17]. For example, Mechtcherine [11] found a positive correlation between the roughness, fractal dimension of crack surfaces, and heterogeneity of concrete based on uniaxial tensile tests. Additionally, it was observed that a higher loading rate resulted in a smoother crack surface. Li et al. [16] observed that the crack area and fractal dimension of concrete were relatively less affected by specimen size and shape under high-temperature conditions, whereas the total crack length and average crack width showed more pronounced dependence on elevated temperatures. Yan et al. [18] discovered that the crack surface exhibited significant fractal characteristics and noted that the increase in fractal dimension was attributed to a higher water–cement ratio and a larger coarse aggregate size. Xu et al. [19] observed that the fractal dimension of the crack surface gradually increased with the increasing number of freeze–thaw cycles for dredged concrete, indicating a gradual roughening of the crack surface. Erdem and Blankson [20] noted that the roughness of crack surfaces increased when the coarse aggregate changed from smooth and rounded to sharp and angular. Ma et al. [21] reported that the fractal dimension of the crack network gradually decreased from a range of 2–3 to 2 when concrete columns transformed from being unconstrained to being constrained with reinforced fiber materials. Based on the existing research, it is evident that concrete crack surfaces are usually irregular with inherent roughness. Moreover, morphological variations in crack surfaces may arise due to different conditions. To accurately analyze the evolution mechanism of tunnel lining leakage, it is necessary to conduct a thorough analysis of the morphological characteristics of crack surfaces based on the actual conditions of concrete lining.

In fact, partial concrete tunnel linings experience a triaxial stress state during normal operations. Under accidental loads, concrete may crack due to high stress, leading to water leakage in the tunnel lining. However, existing studies on the morphological characteristics of concrete crack surfaces often overlook the influence of stress conditions, resulting in disparities between research results and actual engineering situations. The lack of existing research can obscure the mechanisms behind water leakage evolution, leading to ambiguity when developing relevant prevention and control measures.

To address this issue, concrete commonly used in tunnels was prepared, and triaxial loading tests were conducted under different confining pressures. Subsequently, the morphological characteristics of crack surfaces were analyzed using 3D point cloud data, and the influence mechanism of confining pressure on crack surface morphology was further revealed considering the concrete microstructure. Finally, an explanation of how concrete cracks, formed under varying confining pressures, impact leakage in tunnel linings is illustrated from the perspective of fractal dimensions. The research results can provide guidance for understanding the water leakage evolution mechanisms in linings in tunnels and other underground engineering structures.

## 2. Triaxial Loading Test

### 2.1. Test Procedure

The concrete was prepared based on the materials commonly used in Chinese tunnels and the specification for mix proportion design of ordinary concrete [22]. The material ratios are shown in Table 1.

**Figure 1:**
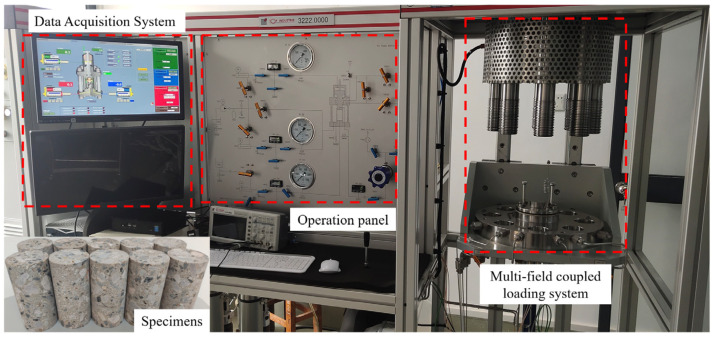
Specimens and test device.

The poured concrete cube blocks with a side length of 150 mm were cured in a standard curing room at 20 °C and 95% relative humidity for 28 days after removing formworks. After curing, the blocks were processed into cylindrical specimens with dimensions of φ50 × 100 mm. These specimens were subjected to uniaxial loading and triaxial loading tests at confining pressures of 2, 4, 6, 10, and 20 MPa, utilizing a multiphase and multifield coupled test system for rock and soil. The system integrates a multifield coupled loading system, an operation panel, and a data acquisition system. It demonstrates maximum pressure capacities of 500 MPa in axial loading and 100 MPa in confining pressure, with exceptional precision metrics including 0.01 MPa stress control accuracy at 1 kPa resolution, and strain measurement accuracy of 1 μm with 0.1 μm resolution. The specimens and loading device are shown in Figure 1. Finally, point cloud data of concrete crack surfaces were acquired using a handheld 3D laser scanning device, which is illustrated at the beginning of Section 3.

### 2.2. Stress–Strain Curves

**Figure 2:**
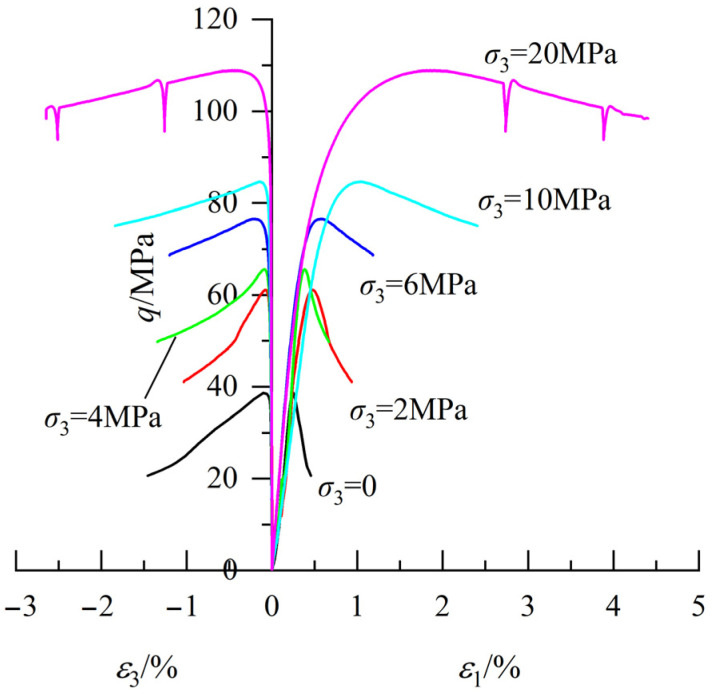
Stress–strain curves of concrete.

The stress–strain curves of concrete under different confining pressures are shown in Figure 2. It should be noted that one representative stress–strain curve is presented per condition to avoid redundancy. In this figure, *ε*_1_ and *ε*_3_ are the axial and lateral strains, respectively, and the deviatoric stress is calculated using Equation (1).

(1) $$q=\sigma_{1}-\sigma_{3}$$

where *q* is the deviatoric stress, and *σ*_1_ and *σ*_3_ are the axial stress and confining pressure, respectively.

The strength of concrete exhibits a positive correlation with confining pressure, accompanied by a gradual transition in failure mode from brittle to ductile as pressure increases, which aligns with existing test results [23]. The compressive strength under uniaxial loading ranges from 37.9 to 40.5 MPa and increases to 106.1–110.6 MPa at 20 MPa confinement. Brittle and ductile failures are characterized by a sudden decrease and a gradual decrease in deviatoric stress, respectively, after reaching peak stress. As can be seen in this figure, under zero confining pressure (*σ*_3_ = 0), the axial strain increases from 0.25% to 0.46% as deviatoric stress in the post-peak stage decreases from 38.7 MPa to 20.6 MPa, corresponding to a 47% reduction in deviatoric stress and an 84% increase in axial strain. When the confining pressure is 20 MPa, the axial strain increases from 1.91% to 4.41% as the deviatoric stress decreases from 108.9 MPa to 98.5 MPa, corresponding to a 10% reduction in deviatoric stress and a 131% increase in axial strain.

In addition, a substantial difference in transverse deformation is observed before and after the peak stress, characterized by a slow increase in transverse deformation before the peak stress and a significant increase afterward. Taking the stress–strain curve at 6 MPa confinement shown in this figure as an example, the transverse strain and corresponding axial strain are −0.20% and 0.57%, respectively, when the deviatoric stress reaches the peak. When loading stops, the transverse strain and corresponding axial strain are −1.20% and 1.18%, respectively. Compared to the states corresponding to peak stress, the lateral strain and axial strain increase by 500% and 107%, respectively, when the test ends. The difference in the transverse deformation behavior before and after the peak stress is related to the evolution of internal microcracks. Before reaching the peak stress, the internal structure of concrete remains relatively intact, and microcracks do not produce significant shear expansion. The transverse strain in this phase mainly comes from the transverse deformation of the concrete matrix due to Poisson’s effect. The main internal structure within the concrete is destroyed after the peak stress is reached, and shear fractures begin to form. Microcracks exhibit volume expansion during shear friction, resulting in a notable increase in the transverse strain of concrete.

### 2.3. Crack Characteristics

**Figure 3:**
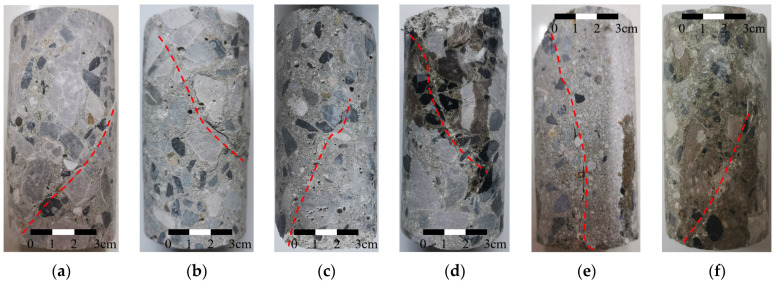
Morphologies of cracks on specimen surfaces under different confining pressures: (**a**) 0 MPa; (**b**) 2 MPa; (**c**) 4 MPa; (**d**) 6 MPa; (**e**) 10 MPa; (**f**) 20 MPa.

**Figure 4:**
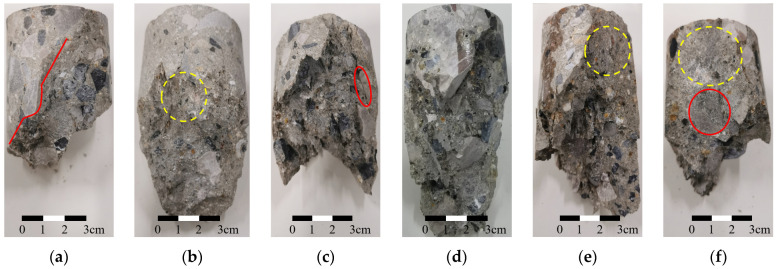
Crack surface morphologies for specimens with different confining pressures: (**a**) 0 MPa: cracks bypass coarse aggregates (red line); (**b**) 2 MPa: uneven crack surface in concrete matrix (yellow circle); (**c**) 4 MPa: preferential cracking at cement–aggregate interface (red circle); (**d**) 6 MPa; (**e**) 10 MPa: flatter crack surface (yellow circle); (**f**) 20 MPa: crack morphology aligns with coarse aggregate shape (red circle), with matrix fracture surface nearly flat (yellow circle).

The characteristic analysis of concrete cracks can be conducted from two perspectives: the crack morphology on the specimen surface and the crack surface morphology. The former can be employed to observe the failure mode of concrete, while the latter can be utilized to analyze the morphological characteristics of concrete crack surfaces. Figure 3 and Figure 4 illustrate the crack morphology on the specimen surface and the crack surface morphology, respectively. In Figure 3, the red dashed line indicates the location and shape of visible cracks on the specimen surfaces.

After the specimens fail, notable inclined cracks appear on the surfaces, indicating that mainly shear failure occurs in the specimens. Compared with coarse aggregate alone, the bonding surface between cementitious materials and coarse aggregate is more prone to failure. This is evident as cracks propagate along the bonding surface, as shown in Figure 3b and Figure 4c. The presence of coarse aggregate influences the characteristics of shear cracks in concrete. The crack does not follow a straight path but exhibits bending when it encounters coarse aggregate on its development path. The reason for this phenomenon is that the crack propagates along the area with lower shear strength, resulting in a change in the direction of crack extension, as shown in Figure 3d. After bypassing the coarse aggregate, the crack resumes its original expansion direction, as indicated by the solid line in Figure 4a. In addition, the development of cracks along the interfaces between coarse aggregates and cementitious materials results in a noticeable similarity between the crack morphology and the shape of the coarse aggregate. As indicated by the red circle in Figure 4f, the crack surface is relatively flat around the coarse aggregates due to their flat shape.

Excluding the influence of coarse aggregates, the crack surface of concrete seems to gradually flatten with an increase in confining pressure. At a confining pressure of 2 MPa, the crack surface of the concrete matrix is slightly rough, as indicated by the yellow circle in Figure 4b. The roughness of the crack surface decreases when the confining pressure increases to 10 MPa, as indicated by the yellow circle in Figure 4e. Under a confining pressure of 20 MPa, the fracture surface of the concrete matrix is almost planar, as indicated by the yellow circle in Figure 4f. The roughness of the concrete crack surface may be related to the constraint degree of crack development. In the absence of confining pressure, concrete can deform unconstrainedly, allowing microcracks near the crack surface to develop freely. This unrestricted crack development leads to an increase in the roughness of crack surface. With an increase in confining pressure, the constraint on concrete deformation increases, and the development of microcracks near the fracture surface is restricted, causing the crack surface to become smoother.

## 3. Morphological Characteristics of Crack Surfaces

The morphology of crack surfaces near coarse aggregates is mainly influenced by the shape of the aggregate particles. It is affected less by the shape of coarse aggregate particles in other regions and more by external factors, such as confining pressure. When conducting seepage analysis within tunnel linings, it is possible to determine the crack morphologies near coarse aggregates based on their shapes. However, there is still significant uncertainty about characterizing the crack morphologies in areas without coarse aggregate. Therefore, this research focuses on analyzing the morphological characteristics of crack surfaces in areas with non-coarse aggregate.

**Figure 5:**
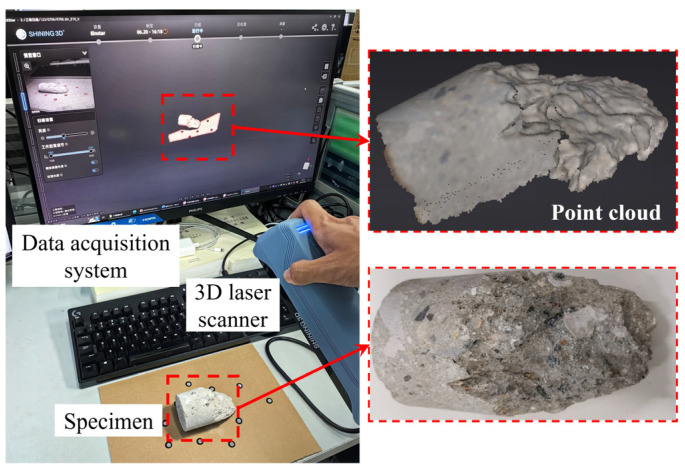
Schematic diagram of 3D laser scanning.

By using a laser scanner device to emit and capture laser beams, it is possible to determine the three-dimensional coordinates of a point by calculating the differences in time and angle between the emitted and reflected laser beams. Through continuous scanning, a series of points representing the surface of an object can be obtained; this is also known as a point cloud. The scanning system used in this study was the Einstar handheld 3D scanner developed by Shining 3D Tech Co., Ltd (Hangzhou, China). This system employs VCSEL-based infrared invisible-light technology for scanning. It can scan at a rate of 14 frames per second with a working distance range from 16 cm to 140 cm. The point spacing in the scanned point cloud ranges from 0.1 mm to 3 mm. A scanning accuracy of 0.1 mm was chosen to obtain detailed morphological features of crack surfaces and avoid computational difficulties caused by dense data points. The 3D laser scanning process is shown in Figure 5.

It can be seen from this figure that there is an angle between the XY plane and the crack surface. This misalignment presents challenges in directly utilizing the point cloud data to analyze the morphological characteristics of crack surfaces. To address this issue, the point cloud data were imported into CloudCompare V2.13.alpha for coordinate adjustment [24]. This adjustment aligns the crack surface with the XY plane.

**Figure 6:**
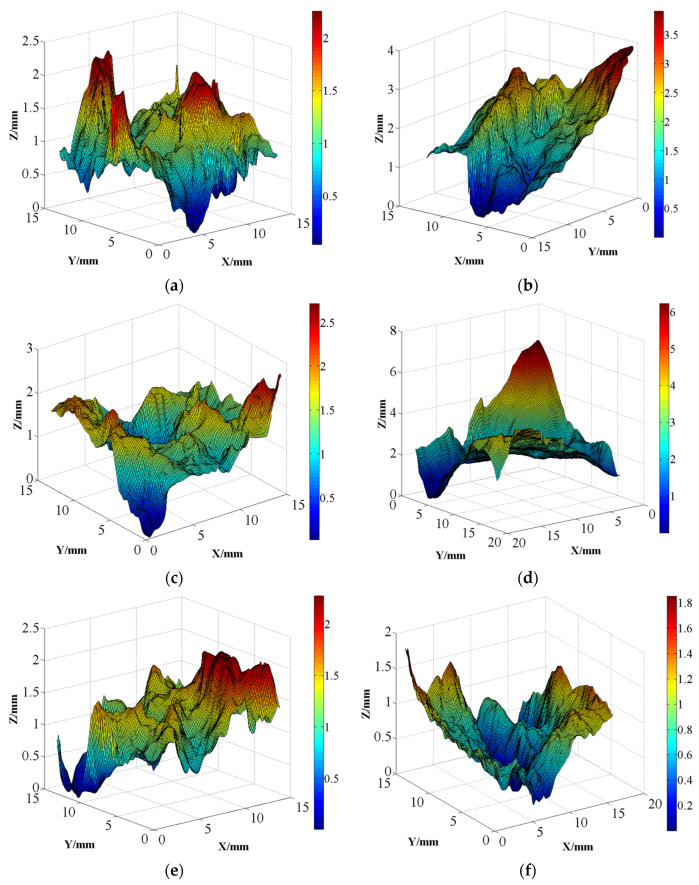
Reconstructed crack surface morphology for specimens under different confining pressures: (**a**) 0 MPa; (**b**) 2 MPa; (**c**) 4 MPa; (**d**) 6 MPa; (**e**) 10 MPa; (**f**) 20 MPa.

The specific steps involved using the plane adaptation function built into the software to identify the plane containing the surface and adjusting the coordinates of points in the point cloud. After coordinate adjustment, the point cloud was imported into MATLAB R2024b (Natick, MA, USA) to reconstruct the concrete crack surfaces. Subsequently, the analysis of the morphological characteristics of the crack surfaces was conducted, including determining the asperity height, inclination angle, and asperity strike, as well as evaluating their fractal dimension. To prevent excessively small crack surface areas from adversely influencing the analysis results, concrete crack surfaces with dimensions of approximately 15 mm in both the *x* and *y* directions were used for morphological feature analysis. The concrete crack surfaces, reconstructed in MATLAB R2024b using 3D laser scanning results, are depicted in Figure 6. To avoid redundancy, only one representative image is presented per condition.

As observed in this figure, concrete crack surfaces exhibit noticeable undulations rather than being flat. For example, when a specimen is subjected to uniaxial loading, there is a significant protrusion in the center of the crack surface and a considerable depression at the edges. Additionally, the concrete crack surface appears to fluctuate irregularly and with a degree of randomness. Despite the apparent randomness in the undulation of crack surfaces, their height tends to flatten as the confining pressure increases. The maximum heights of crack surfaces are approximately 4.0(±0.8), 2.7(±0.7), 2.4(±0.5), and 2.0(±0.6) mm for specimens subjected to confining pressures of 2, 4, 10, and 20 MPa, respectively. The extent of undulation in the concrete crack surface appears to be related to the development degree of microcracks. In the case of lower confining pressure, the concrete near the crack surface can develop microcracks freely, resulting in greater height variations on the crack surface. With the increase in confining pressure, the development of microcracks near the crack surface becomes restricted, leading to a decrease in the unevenness of crack surface.

### 3.1. Asperity Height

The height distribution of asperities is an important parameter characterizing the characteristics of concrete crack surfaces. To obtain this distribution, the 3D point cloud data after coordinate adjustment were meshed on the XY plane, with each grid representing an asperity. Each asperity has a length and width of 0.1 mm. The height of an asperity equals the average value of the *z*-coordinates of its four vertices. Through calculations, it is possible to obtain the occurrence frequency for different heights and the probability density functions.

The distribution characteristics of asperities can be further analyzed from the perspectives of skewness and kurtosis [25]. Skewness is a parameter characterizing the symmetry of data distribution and can be calculated using Equation (2) for ungrouped data. A skewness value of 0 indicates a symmetrical data distribution, and the data follows a standard normal distribution. Data with skewness greater than 0 follows a right-skewed distribution, whereas data with skewness smaller than 0 follows a left-skewed distribution. The degree of skewness is lower the closer its value is to 0. If the absolute value of skewness falls between 0.5 and 1, the data is moderately skewed, while it is highly skewed if the absolute value is greater than 1.

(2) $$\mathrm{skew}=\frac{\frac{1}{n}\sum_{i=1}^{n}{\left(h_{i}-\bar{h}\right)}^{3}}{{\left(\sqrt{\frac{1}{n}\sum_{i=1}^{n}{\left(h_{i}-\bar{h}\right)}^{2}}\right)}^{3}}$$

where *n* is the number of asperities, and $\bar{h}$ is the average asperity height.

Kurtosis can be calculated using Equation (3). The kurtosis equals 3 when the data follows a standard normal distribution, and the corresponding distribution can also be named mesokurtic distribution. The data distribution is more concentrated than the normal distribution at kurtosis values significantly exceeding 3. In this case, the peak of the probability distribution function is high and narrow, and this kind of distribution can be named leptokurtic distribution. Conversely, if the kurtosis is significantly less than 3, the data distribution is flatter than the normal distribution and considered more discrete. This data distribution can be named platykurtic distribution.

(3) $$\mathrm{kurt}=\frac{\frac{1}{n}\sum_{i=1}^{n}{\left(h_{i}-\bar{h}\right)}^{4}}{{\left(\frac{1}{n}\sum_{i=1}^{n}{\left(h_{i}-\bar{h}\right)}^{2}\right)}^{2}}$$

**Figure 7:**
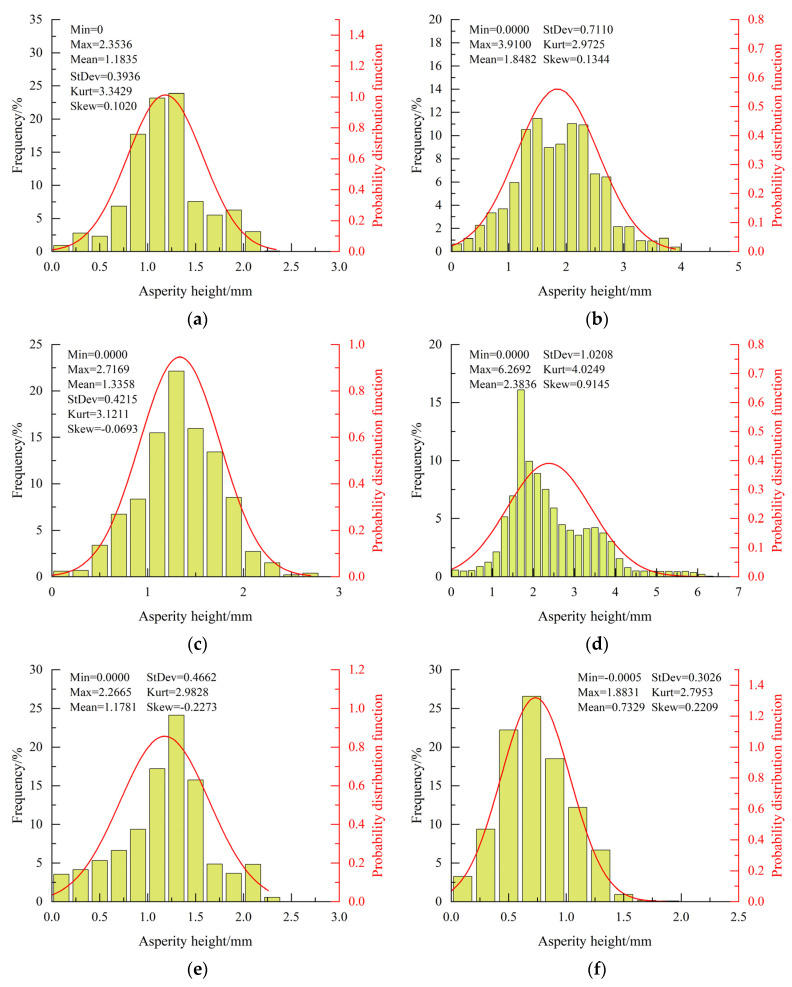
Height distribution of asperities for specimens under different confining pressures: (**a**) 0 MPa; (**b**) 2 MPa; (**c**) 4 MPa; (**d**) 6 MPa; (**e**) 10 MPa; (**f**) 20 MPa. The height statistics include minimum (Min), maximum (Max), mean height (Mean), standard deviation (StDev), kurtosis (Kurt), and skewness (Skew).

Figure 7 illustrates the frequency and distribution of asperity height under different confining pressures. It should be noted that only one representative image is presented per condition; others are not illustrated to avoid redundancy. Except for the specimen with a confining pressure of 6 MPa, the skewness of asperity heights under other conditions falls within the range of ±0.25, showing a low skewness distribution characteristic. Moreover, as the confining pressure increases, the peak of the probability distribution function for asperity heights gradually decreases, transitioning from a leptokurtic distribution to a platykurtic distribution. For specimens subjected to uniaxial loading and triaxial loading with confining pressures of 4, 10, and 20 MPa, the kurtosis of asperity heights is about 3.3(±0.4), 3.1(±0.5), 3.0(±0.3), and 2.8(±0.4), respectively.

The results clearly indicate that the distribution of asperity height significantly deviates from a normal distribution. To further evaluate the data distribution, statistical tests were performed using multiple candidate distributions, including the chi-square distribution. However, the results consistently demonstrated that concrete crack surfaces do not follow any of the tested distributions. This suggests that the distribution of the asperity heights of concrete crack surfaces exhibits strong randomness. Due to this finding, confidence intervals and *p*-values are not reported in the analysis. The statistical characterization of asperity height distributions on tensile crack surfaces also indicates distinct non-normal distribution patterns, predominantly manifesting as skewed or platykurtic distributions [26].

### 3.2. Inclination Angle

**Figure 8:**
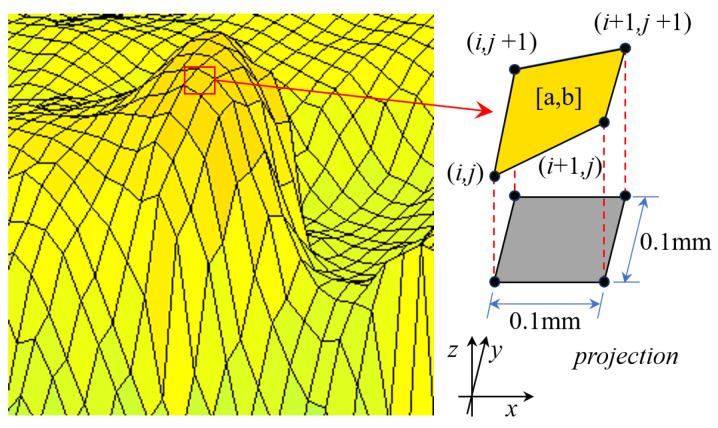
Schematic diagram of asperity and node numbering.

The inclination angle can be used to reflect the fluctuation in crack surfaces. A greater inclination angle corresponds to a steeper fracture surface. To facilitate expression, the asperities and nodes are numbered. The notation [a, b] represents the sequence of asperities in both the *x* and *y* directions. Similarly, the notation (*i*, *j*) is used to denote the order of nodes in the *x* and *y* directions, as depicted in Figure 8. Equations (4) and (5) illustrate the calculation methods of the vector components for an asperity in the *x* and *y* directions, and Equation (6) presents the method for calculating the unit normal vector of an asperity.

(4) $$\vec{v_{x}}=\{(x_{i,j},y_{i,j},z_{i,j})-(x_{i+1,j},y_{i,j},z_{i+1,j})\}$$

(5) $$\vec{v_{y}}=\{(x_{i,j},y_{i,j},z_{i,j})-(x_{i,j},y_{i,j+1},z_{i,j+1})\}$$

(6) $${\vec{v}}_{norm}=\frac{\vec{v_{x}}\times \vec{v_{y}}}{\left||\vec{v_{x}}\times \vec{v_{y}}|\right|}$$

where *x*, *y*, and *z* represent the node coordinates, the subscripts *i* and *j* are the node numbers in the *x* and *y* directions, and $\left||\vec{v_{x}}\times \vec{v_{y}}|\right|$ is the second-order norm of $\vec{v_{x}}\times \vec{v_{y}}$.

The inclination angle is the angle between the normal vector of an asperity and the positive *z*-axis direction and can be calculated using Equation (7).

(7) $$\alpha =\arccos\frac{{\vec{v}}_{norm}\cdot \vec{e_{z}}}{\left|{\vec{v}}_{norm}|\cdot |\vec{e_{z}}\right|}$$

where $\vec{e_{z}}$ is the unit vector in the *z*-direction, $\vec{e_{z}}=(0,0,1)$.

**Figure 9:**
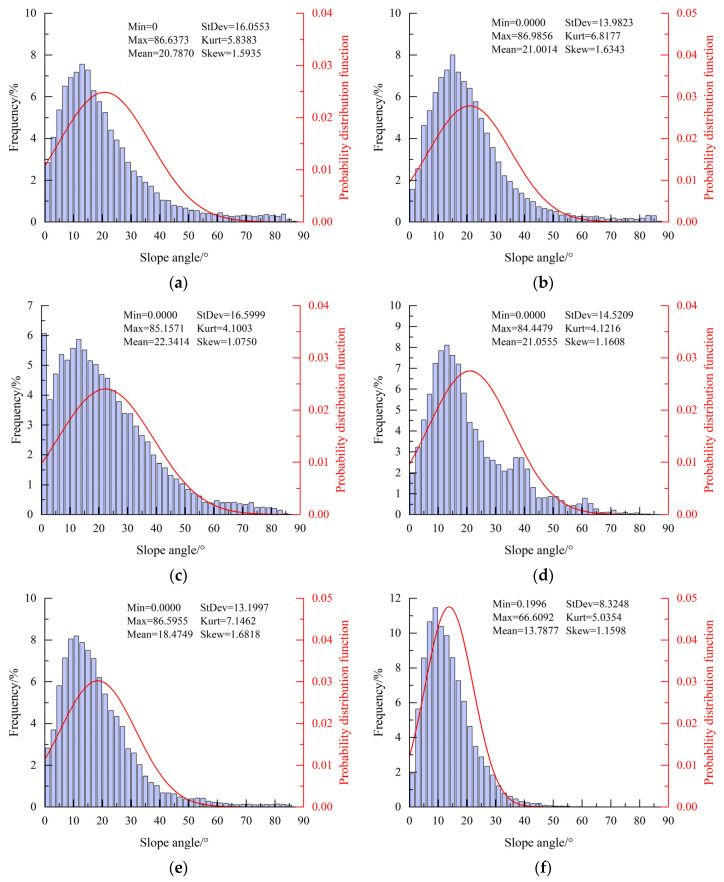
Asperity inclination angle distribution for specimens under different confining pressures ((**a**): 0 MPa; (**b**): 2 MPa; (**c**): 4 MPa; (**d**): 6 MPa; (**e**): 10 MPa; (**f**): 20 MPa). The slope angle statistics include minimum (Min), maximum (Max), mean height (Mean), standard deviation (StDev), kurtosis (Kurt), and skewness (Skew).

Figure 9 depicts the frequency and distribution of asperity inclination angles under different confining pressures. To avoid redundancy, only one representative image is presented for each condition. Notably, the asperity inclination angles mainly fall within the range of low angles, and the inclination angle distribution has obvious skewness and leptokurtic characteristics, indicating a deviation from normal distribution. Across different confining pressures, calculations reveal that over 85% of the total asperities have inclination angles of less than 40°. The skewness and kurtosis values of the inclination angle distribution are in the ranges of 1.0–1.7 and 4.0–7.0, respectively. Furthermore, the inclination angle with the highest frequency tends to decrease as the confining pressure increases. For the data shown in this figure, the predominant inclination angles are 15(±1)°, 13(±1)°, 11(±1)°, and 9(±1)° under confining pressures of 2, 6, 10, and 20 MPa, respectively. A similar distribution analysis to that mentioned in Section 3.1 was conducted for the inclination angle distribution, which also showed no conformity to the tested distributions. Consequently, confidence intervals and *p*-values are omitted for brevity. Notably, statistical characterization of inclination angle distributions on tensile crack surfaces also indicates distinct non-normal distribution patterns, predominantly manifesting as skewed or platykurtic distributions [26].

### 3.3. Asperity Strike

The asperity strike refers to the angle between the normal vector projection of an asperity in the XY plane and in the positive *x*-axis direction. It can be calculated using Equation (8). A higher presence of asperities with the same strike indicates a greater inclination tendency of the crack surface in that direction. In the presence of regular undulations on the crack surface, a notable degree of symmetry in asperity strike distribution can be observed.

(8) $$\beta =\arccos\frac{{\vec{v}}_{proj}\cdot \vec{e_{x}}}{\left|{\vec{v}}_{proj}|\cdot |\vec{e_{x}}\right|}$$

where $\vec{e_{x}}$ is the unit vector in the *x* direction, $\vec{e_{x}}=(1,0,0)$, ${\vec{v}}_{proj}$ is the projection of the normal vector in the XY plane, and |·| is the modulus of a vector.

**Figure 10:**
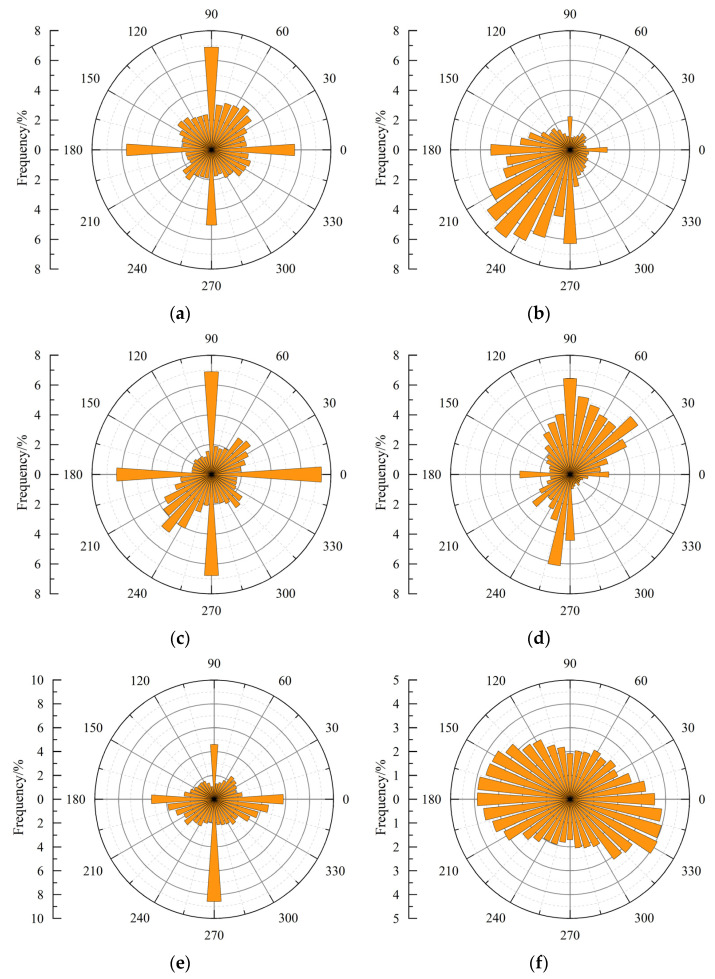
Asperity strike distribution for specimens under different confining pressures: (**a**) 0 MPa; (**b**) 2 MPa; (**c**) 4 MPa; (**d**) 6 MPa; (**e**) 10 MPa; (**f**) 20 MPa.

Figure 10 displays the occurrence frequencies of asperity strikes under various confining pressures. It should be noted that each condition only displays one representative image and others are not illustrated to avoid redundancy. In this figure, the circumferential and radial values represent strikes and corresponding occurrence frequencies, respectively. Observations reveal an uneven and discernible trend in the asperity strike distribution. For example, as can be seen in the figure, most asperity strikes cluster between 175° and 275° under a confining pressure of 2 MPa, whereas they predominantly fall within the range of 25° to 125° under a 6 MPa confining pressure. According to Figure 6b,d, the concrete crack surface exhibits a pronounced inclination towards the positive *y*-axis and the positive *x*- and *y*-axes, respectively. Moreover, as the confining pressure rises, the randomness of asperity strikes increases and the strike tendency decreases. According to the data shown in this figure, for specimens subjected to confining pressures of 2, 4, 6, 10, and 20 MPa, the number of strikes exceeding 5% is 7, 4, 4, 3, and 0, respectively. Among them, all strikes fall within 175–275° when the confining pressure is 2 MPa, while only three directions are within 25–95° when the confining pressure is 6 MPa. Existing studies on crack surface morphologies have consistently observed anisotropic asperity strike distributions [26]. This phenomenon arises because prominent surface irregularities such as large humps or hollow spaces cause multiple grid elements to share identical orientations, resulting in non-uniform frequency distributions of asperity strike.

### 3.4. Fractal Dimension

Geometric fractals represent miniature geometric entities that contain structural features of geometric bodies. The fractal dimension serves as a statistical metric quantifying the extent to which a geometric fractal occupies space. The box-counting method was used to further study the morphological characteristics of concrete crack surfaces by calculating their fractal dimensions. The fundamental principle behind the box-counting method involves covering a crack surface with cubic boxes [26]. The following equation illustrates the relationship between the total number of cube boxes required to cover a crack surface and the length of the cube side.

(9) $$N(\epsilon )\sim 1/\epsilon^{D}$$

where *ϵ* is the side length of the cube box, *N*(*ϵ*) is the total number of boxes required to cover a crack surface, and *D* is the fractal dimension.

By projecting the crack surface into the XY plane and meshing it, the crack surface can be divided into small regions with dimensions of *ϵ*. Starting from the origin, the order of vertex occurrences within these subregions along the *x* and *y* axes are marked with *k* and *l*, respectively. After dividing the subregions, the cube box with a side length of *ϵ* is used to cover the crack surfaces. The number of cube boxes required to cover a subregion is calculated using Equation (10).

(10) $$N_{k,l}=INT\{\left[\max(z_{k,l},z_{k+1,l},z_{k,l+1},z_{k+1,l+1})-\min(z_{k,l},z_{k+1,l},z_{k,l+1},z_{k+1,l+1})\right]/\epsilon +1\}$$

where *INT* is the rounding function, i.e., returning the maximum integer not exceeding the real number input.

The total number of cube boxes required to cover a crack surface can be calculated using the following equation:

(11) $$N(\epsilon )=\sum_{k,l=1}^{n-1}N_{k,l}$$

Further, the fractal dimension of a crack surface can be calculated using Equation (12).

(12) $$D=\underset{\epsilon \to 0}{\lim}(\log N(\epsilon )/\log(1/\epsilon ))$$

**Figure 11:**
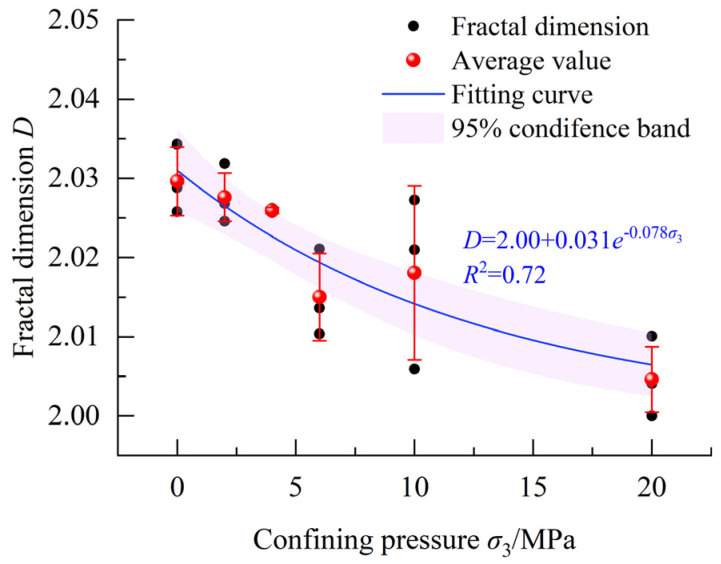
Fractal dimensions of crack surfaces under different confining pressures.

Figure 11 illustrates the fractal dimensions of concrete crack surfaces under different confining pressures. Notably, the fractal dimension of the concrete crack surface progressively decreases as the confining pressure increases. For instance, under uniaxial loading and triaxial loading with a 20 MPa confining pressure, the fractal dimensions of crack surfaces fall within the ranges of 2.025–2.035 and 2.000–2.010, respectively. The corresponding average values are 2.030 and 2.005. It should be noted that the influence of coarse aggregate on fractal dimensions is not considered in this study. When considering the influence of coarse aggregate, the fractal dimensions of a crack surface can be larger. For example, Fu et al. [27] reported fractal dimensions up to 2.5 for concrete crack surfaces in three-point bending tests, significantly higher than those in our experiments.

The experimental results indicate that the fractal dimensions of the crack surfaces appear to gradually decrease with increasing confining pressure. Considering that the fractal dimension of a plane is 2.00, the negative exponential function shown in Equation (13) can be employed to perform regression analysis on the complete dataset.

(13) $$D=2.00+Ae^{B\sigma_{3}}$$

where *A* and *B* are constants and determined through the regression analysis of the experimental data.

The values of *A* and *B* along with their standard deviations, 95% confidence intervals, *p*-values, and *R*^2^ can be obtained through regression analysis, as shown in Table 2. The *p*-values were derived from hypothesis tests where either *A* = 0 or *B* = 0 was set as the null hypothesis. The calculated results demonstrate that the confidence interval for neither *A* nor *B* includes zero, with both *p*-values being less than 0.05. This confirms the statistical significance of the parameters, establishing a negative exponential relationship between the fractal dimension of the crack surface and the confining pressure.

## 4. Discussion

### 4.1. Influence Mechanism of Confining Pressure on Features of Concrete Crack Surfaces

It is generally recognized that microcracks initiate and propagate progressively in concrete under loading conditions, eventually coalescing into macrocracks and leading to structural failure. The crack behavior of concrete is intrinsically governed by its material composition [28], which influences the crack characteristics of concrete through modifications of its micromechanical properties. Investigating crack evolution across microscales provides critical insights into the development mechanisms of macrocracks. Given recent advancements in characterization techniques for material microstructure and mechanical properties [29], scanning electron microscopy is introduced to examine the characteristics of microcracks in concrete, aligning with the research objectives.

**Figure 12:**
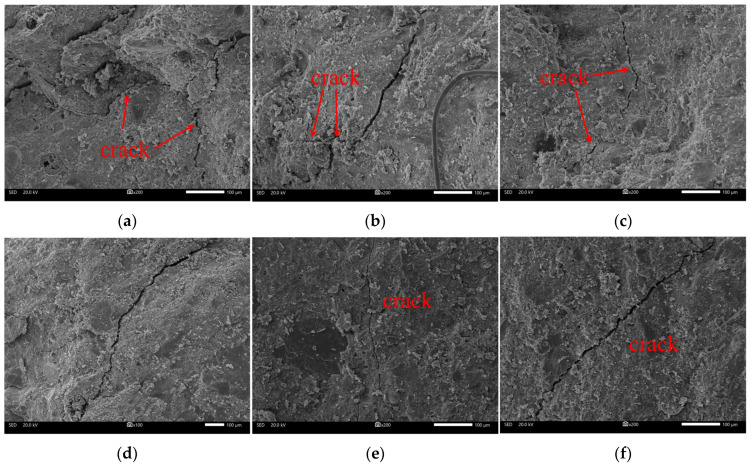
Characteristics of concrete microcracks under different confining pressures: (**a**) 0 MPa; (**b**) 2 MPa; (**c**) 4 MPa; (**d**) 6 MPa; (**e**) 10 MPa; (**f**) 20 MPa.

Using high-energy electron beams to scan samples, scanning electron microscopy can be employed to observe the microscopic morphology of materials by collecting the excited physical information. Figure 12 illustrates the microcracks near the concrete crack surface. According to this figure, there is a relationship between the microcrack morphology and the confining pressure. This relationship is primarily characterized by a gradual reduction in crack number with the increase in confining pressure, accompanied by an increase in crack flatness. In the case of uniaxial loading, the microcracks exhibit pronounced twists, as depicted in Figure 12a. Under a confining pressure of 20 MPa, the cracks within the concrete matrix are almost linear, and their flatness increases significantly.

According to Figure 4 and Figure 12, it can be concluded that the roughness of the concrete crack surface is related to the presence or absence of restrictions on the development of microcracks near the crack surface. Concrete is a composite material formed through the mixing of coarse aggregate, fine aggregate, cementitious materials, admixtures, etc. Affected by the stochastic distribution of aggregate particles, there is inherent variability in the distribution of weak structural planes inside concrete, which in turn causes microcracks to appear in random locations and directions within the concrete during loading. When subjected to a high load, microcracks gradually initiate and propagate within the concrete, ultimately coalescing to form macrocracks.

**Figure 13:**
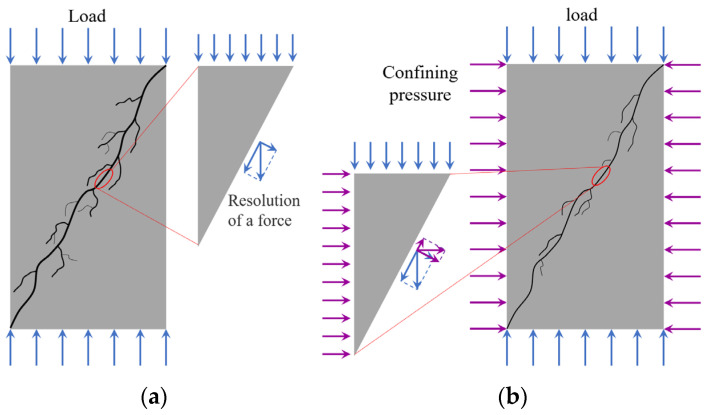
Schematic diagram for confining pressure impact on concrete crack surface roughness: (**a**) low confining pressure; (**b**) high confining pressure.

At low confining pressures, the concrete matrix near the crack surface experiences small constraints, allowing microcracks to develop freely, as shown in Figure 13a. The interconnection of microcracks near the crack surface can lead to a detachment of concrete between these microcracks and the crack surface, leading to an increase in crack surface roughness. As the confining pressure increases, the compressive stress borne by the concrete around the crack surface increases, as shown in Figure 13b. The heightened constraints limit the extent of microcrack development in this region, resulting in a smoother crack surface with a lower fractal dimension.

### 4.2. Influence of Concrete Cracks Formed Under Various Confining Pressures on Tunnel Lining Leakage

Under the same conditions, alterations in the roughness of the concrete crack surface can influence the probability of leakage in the tunnel lining. This is because changes in crack surface roughness can lead to variations in the width of the seepage channel, which in turn affects water flow within cracks. Existing research findings suggest that there is a negative correlation between the seepage flow rate within cracks and the roughness of the crack surface [30]. Wang et al. [31] systematically investigated crack-induced permeability changes in sandstone. They employed laser scanning confocal microscopy to analyze crack surfaces across sandstone with varying grain sizes, using the 3D fractal dimension as the primary roughness metric. Flow tests were then conducted via a custom-designed seepage flow system, and a seepage model for sandstone with a single rough fracture was further established, as shown in Equation (14). The model demonstrated strong agreement with the experimental data, confirming its validity [31].

(14) $$Q=10^{-3}(17.41-6.40D)\frac{b^{3}J}{\mu }$$

where *Q* is the seepage rate of a crack with unit width (m^3^/s); *D* is the fractal dimension; *b* is the crack opening width (m); *μ* is the dynamic viscosity coefficient (Pa·s), which equals 100.5 × 10^−5^ Pa·s for water at one atmospheric pressure and 20 °C; and *J* is the hydraulic gradient, considering the influence of fluid density and gravitational acceleration (Pa/m).

In practical engineering, materials with permeability coefficients (*K*) of less than 0.001 m/d (K < 1.16 × 10^−6^ cm/s) are classified into impermeable layers [32], such as clay layers, peat layers, and impermeable bedrock. In addition, the permeability coefficient has the following relationship with the seepage rate:

(15) $$K=\frac{Q}{A_{a}i_{h}}$$

where *K* is the permeability coefficient, m/s; *A_a_* is the discharge area, m^2^; and *i_h_* is the hydraulic gradient within a crack.

According to Equations (14) and (15), the permeability coefficient of concrete containing a single rough crack can be calculated using the following equation:

(16) $$K=10^{-3}(17.41-6.40D)\frac{b^{2}}{\mu }$$

It can be seen from this equation that concrete cracks formed under high confining pressure are more likely to cause water leakage in tunnel linings. This phenomenon arises due to a reduction in the fractal dimension of the crack surface with the increasing confining pressure. Concrete cracks formed under high confining pressure have a higher permeability coefficient under the same conditions, thereby rendering tunnel linings more susceptible to water leakage. It is worth noting that this study’s fractal dimension calculation for concrete crack surfaces does not account for the influence of coarse aggregate. Consequently, the calculated fractal dimensions of concrete fracture surfaces are small. In fact, the fractal dimension of a crack surface can reach values as high as 2.6, and even 3.0 in extreme cases [19,33]. Considering the actual situation of concrete cracks and taking concrete with a crack width of 0.1 mm as an example, the permeability coefficients of concrete are 7.66 × 10^−7^ cm/s and 3.95 ×10^−6^ cm/s when the fractal dimensions of crack surfaces are 2.6 and 2.1, respectively, as calculated using Equation (16). Accordingly, the tunnel lining can be categorized as an impermeable layer and a permeable layer, respectively.

## 5. Conclusions and Research Proposals

### 5.1. Conclusions

1. The strength of concrete specimens is positively related to confining pressure, and specimens in tests mainly exhibit shear failure. In addition, the failure mode gradually transforms from brittle to ductile with the increase in confining pressure. There is a slow and then significant increase before and after reaching the peak stress for transverse deformation in concrete. The presence of coarse aggregate can lead to bending to some extent in shear cracks. Without considering the influence of coarse aggregate, concrete crack surfaces gradually flatten as the confining pressure increases.
2. The distribution of asperities on concrete crack surfaces exhibits strong randomness, and the inclination angle distribution of asperities has obvious skewness and leptokurtic characteristics. In addition, there is an uneven and discernible trend in asperity strike distribution. Statistical analysis reveals that more than 85% of the total asperities have inclination angles of less than 40°. As the confining pressure increases, the kurtosis of asperity height tends to transform from a leptokurtic distribution to a platykurtic distribution, and the randomness of asperity strikes increases. In contrast, some factors gradually decrease, including the inclination angle with the highest frequency and the fractal dimensions of crack surfaces. A negative exponential function can effectively depict the relationship between the fractal dimension of the concrete crack surface and the confining pressure.
3. The number of microcracks within concrete decreases with the increase in confining pressure, while the flatness of these microcracks gradually increases. Under low confining pressure, microcracks within the concrete matrix near crack surfaces can develop freely, further leading to an increase in crack surface roughness. With increasing confining pressure, the heightened constraints limit the extent of microcrack development in the concrete matrix near the crack surface, resulting in a smoother crack surface with a lower fractal dimension. As the fractal dimension decreases with an increase in confining pressure, the concrete cracks formed exhibit higher permeability coefficients, rendering tunnel linings more susceptible to water leakage.

### 5.2. Research Proposals

Underground structures such as tunnels often face groundwater leakage during service. The initiation and propagation of leakage are inherently linked to the development of seepage channels. The characteristics of concrete crack surfaces can be analyzed in order to establish seepage channel models. By integrating theories of seepage flow, dissolution, and erosion, the propagation process of seepage channels can be systematically analyzed, enabling the prediction of leakage evolution and providing critical guidance for the safe operation of underground infrastructure.

Current research on concrete crack surfaces primarily focuses on localized crack characteristics. Future studies can investigate the characteristics of crack surfaces across larger scales. Additionally, the formation and propagation of microcracks in concrete significantly influence the emergence of macrocracks. Further research may explore the nanoscale mechanisms, such as the debonding of C-S-H gels and the cement–aggregate interface, to enhance the understanding of crack propagation and improve leakage prediction models.

## Figures and Tables

**Table 1:** Concrete mix ratio (mass ratio, kg).

P.O 42.5 Cement	Fly Ash	Medium Coarse Sand	Gravel	Water	Water-Reducing Admixture
405	45	690	1165	135	5.8

**Table 2:** Results of the regression analysis.

Parameters	Value	Standard Deviation	95% Confidence Interval	*p*-Value	*R* ^2^
*A*	0.031	0.002	[0.026, 0.036]	0.000	0.72
*B*	−0.078	0.017	[−0.114, −0.043]	0.000	

## Data Availability

The data presented in this study are available on request from the corresponding author. The data are not publicly available due to privacy restrictions.

## Abbreviations

The following abbreviations are used in this manuscript:

3D	three-dimensional
